# Genetic Analysis of the Electrophysiological Response to Salicin, a Bitter Substance, in a Polyphagous Strain of the Silkworm *Bombyx mori*

**DOI:** 10.1371/journal.pone.0037549

**Published:** 2012-05-23

**Authors:** Tetsuya Iizuka, Toshiki Tamura, Hideki Sezutsu, Keisuke Mase, Eiji Okada, Kiyoshi Asaoka

**Affiliations:** 1 Transgenic Silkworm Research Unit, National Institute of Agrobiological Sciences, Tsukuba, Ibaraki, Japan; 2 College of Humanities and Sciences, Nihon University, Setagaya-ku, Tokyo, Japan; 3 Insect–Plant Interaction Research Unit, National Institute of Agrobiological Sciences, Tsukuba, Ibaraki, Japan; AgroParisTech, France

## Abstract

Sawa-J is a polyphagous silkworm (*Bombyx mori* L.) strain that eats various plant leaves that normal silkworms do not. The feeding preference behavior of Sawa-J is controlled by one major recessive gene(s) on the *polyphagous* (*pph*) locus, and several minor genes; moreover, its deterrent cells possess low sensitivity to some bitter substances including salicin. To clarify whether taste sensitivity is controlled by the *pph* locus, we conducted a genetic analysis of the electrophysiological characteristics of the taste response using the polyphagous strain Sawa-J·*lem*, in which *pph* is linked to the visible larval marker *lemon* (*lem*) on the third chromosome, and the normal strain Daiankyo, in which the wild-type gene of *pph* (+*^pph^*) is marked with *Zebra* (*Ze*). Maxillary taste neurons of the two strains had similar dose–response relationships for sucrose, inositol, and strychnine nitrate, but the deterrent cell of Sawa-J·*lem* showed a remarkably low sensitivity to salicin. The F_1_ generation of the two strains had characteristics similar to the Daiankyo strain, consistent with the idea that *pph* is recessive. In the BF_1_ progeny between F_1_ females and Sawa-J·*lem* males where no crossing-over occurs, the *lem* and *Ze* phenotypes corresponded to different electrophysiological reactions to 25 mM salicin, indicating that the gene responsible for taste sensitivity to salicin is located on the same chromosome as the *lem* and *Ze* genes. The normal and weak reactions to 25 mM salicin were segregated in crossover-type larvae of the BF_1_ progeny produced by a reciprocal cross, and the recombination frequency agreed well with the theoretical ratio for the loci of *lem*, *pph*, and *Ze* on the standard linkage map. These results indicate that taste sensitivity to salicin is controlled by the gene(s) on the *pph* locus.

## Introduction

Chemical constituents in plants provide information for determining the host range in phytophagous insects [1], [2]. Among various factors, taste information is key for initiating food intake [1]–[3]. The domesticated silkworm, *Bombyx mori* L., is a monophagous insect that can be raised on fresh mulberry leaves (*Morus alba* L.). Several silkworm feeding stimulants have been isolated from mulberry leaves [4], [5]; among them, sucrose is a powerful feeding stimulant [6], and *myo*-inositol synergizes the effect of sucrose [7]. The sucrose-best and inositol-specific taste neurons are both present on the lateral sensillum styloconicum of the maxillary galea [8]. In contrast, secondary compounds in non-host plants could also be feeding deterrents for host-plant selection by the silkworm. The taste neurons for detecting these chemicals, collectively referred to as deterrent cells, are present in the medial sensillum styloconicum on the maxillary galea [9] as well as in the epipharyngeal sensillum on the ventral side of the labrum [10]. Torii and Morii [11] previously demonstrated the important role of the maxilla in obtaining inhibitory signals to distinguish food plants, in which they reported a silkworm with no maxilla that ate many different types of plant leaves rather than just mulberry leaves. In addition, Ishikawa and Hirao [7] and Kanda [12] showed that inactivation by acid treatment or extirpation of the medial styloconic sensilla made silkworm larvae feed on non-host plant leaves or an artificial diet lacking mulberry leaf powder (LP-1) that normal silkworms will not eat. Although it is possible that other deterrent cells, such as those found in the maxillary palp tip sensilla of *Manduca sexta* larvae [13] or the specialized deterrent cells found in the maxillary styloconic sensilla of *Pieris* caterpillars [14], are present in silkworm, the activation of different deterrent cells produced the same deterrent effect on feeding in other lepidopteran larvae [2], [15], [16].

Despite being largely monophagous, some silkworm strains eat many different types of plant leaves [17]–[19]. One of the most representative strains is Sawa-J, which was bred by Yokoyama [19]. The strain is called “polyphagous” because it eats a broader range of plants [17]. This strain can be raised on the LP-1 artificial diet [12]. A genetic analysis of the polyphagous character of the Sawa-J strain showed that feeding preference behavior is controlled by major gene(s) on a recessive mutant locus and several minor genes on other loci [12], [20]. The gene(s) on the major locus is designated *polyphagous* (*pph*), and the locus has been mapped to 12.9 cM on the third chromosome [21]. Electrophysiological studies using the Sawa-J strain as well as another polyphagous strain have shown that the deterrent cells of these strains in both the medial maxillary styloconic sensillum and the epipharyngeal sensillum are abnormal; i.e., the electrophysiological response against salicin, a bitter tastant for people, is much weaker in these polyphagous strains than in normal strains [22], [23], although the response to strychnine nitrate is similar among silkworm strains [22]. Both salicin and strychnine nitrate stimulate the same deterrent cell in the medial styloconic sensilla on the maxillary galea and deter feeding of normal silkworm larvae [9], [24]. However, the deterrent effects of salicin determined by both the intake of salicin-containing diet 15 h after the fourth ecdysis and the initiation time of the first meal feeding are lower in the polyphagous strains compared to normal strains [23], [24]. The deterrent effect of strychnine nitrate was observed in the Sawa-J strain as well as the normal strains [24].

Thus, the Sawa-J strain exhibits polyphagous feeding and a low sensitivity in the sensory response to some bitter substances, including salicin. However, no direct genetic experiment has shown the relationship between the function of the gene(s) on the *pph* locus and the electrophysiological taste response. To clarify whether the response is controlled only by the gene(s), we performed a genetic analysis using the polyphagous strain, Sawa-J·*lem*, and the normal strain, Daiankyo. The study reported here using the two parent strains and their F_1_ and BF_1_ progeny indicated that the response to salicin is controlled by the gene(s) on single *pph* locus.

## Materials and Methods

### 1. Silkworm Strains

**Figure 1:**
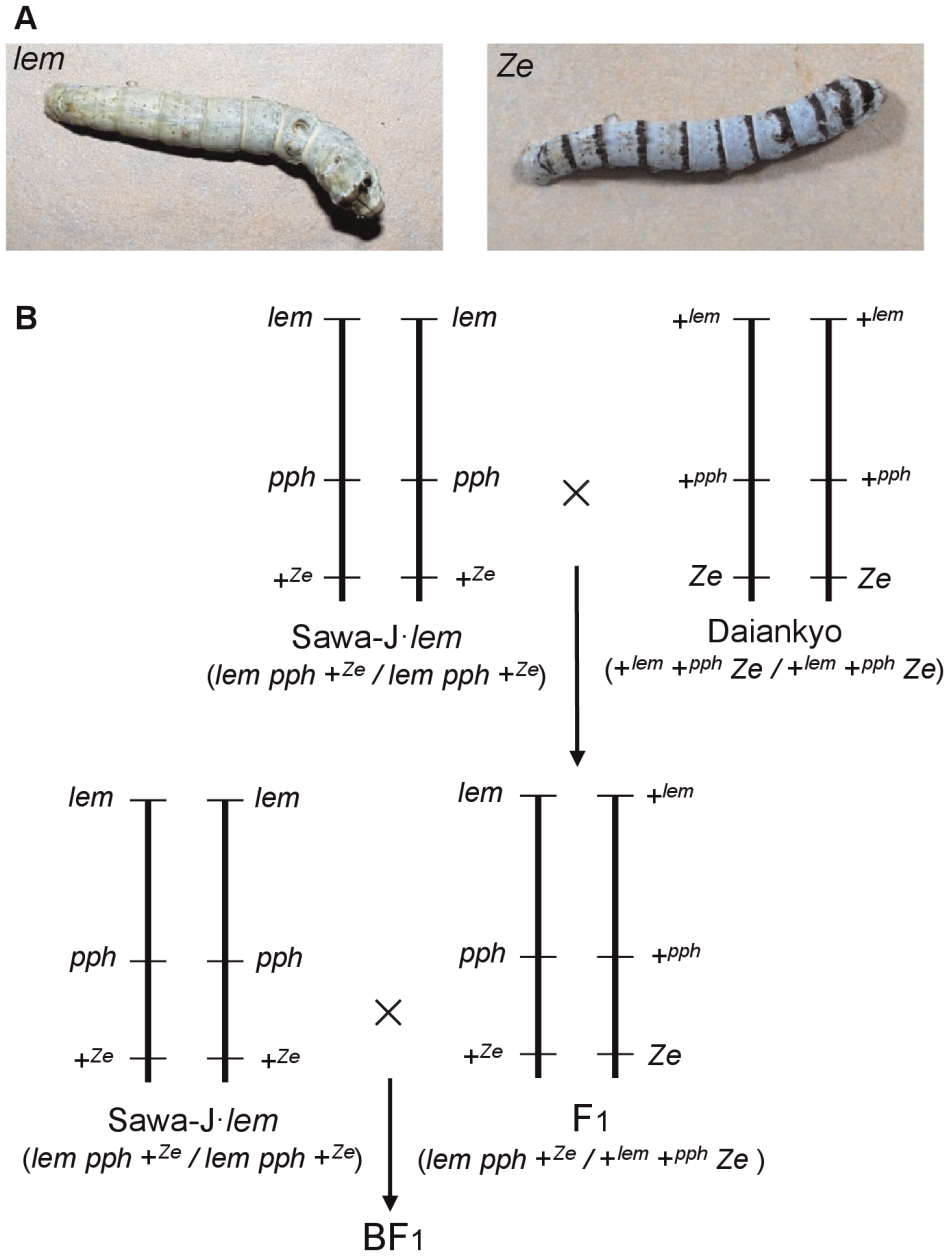
Phenotypes and the crossing scheme of the two strains, Sawa-J·*lem* (*lem*) and Daiankyo (*Ze*). A, Larvae of Sawa-J·*lem* and Daiankyo showing the phenotypes of the marker genes, *lemon* (*lem*) and *Zebra* (*Ze*), respectively. B, Crossing of Sawa-J·*lem* and Daiankyo to obtain F_1_ and BF_1_ larvae for an analysis of electrophysiological response to salicin.

Two strains, Sawa-J·*lem* [21] and Daiankyo (ANJP No.335, NIAS Genebank), were used to construct F_1_ and backcross (BF_1_) generations and to analyze the electrophysiological taste response. The former strain, whose genotype on the third chromosome is *lem pph* +*^Ze^/lem pph* +*^Ze^*, is preserved at the Transgenic Silkworm Research Unit, and the latter, whose genotype is +*^lem^* +*^pph^ Ze/*+*^lem^* +*^pph^ Ze*, is at the Genetic Resources Conservation Research Unit of the National Institute of Agrobiological Sciences, Japan. The phenotypes of *lemon* (*lem*) and *Zebra* (*Ze*) are easily distinguished by their yellow integument and black-striped zebra pattern, respectively, at the larval stage (Fig. 1A). The crossing scheme is shown in Figure 1B. To identify the chromosome controlling the electrophysiological response to salicin, the BF_1_ larvae between the female F_1_ and male Sawa-J·*lem* were examined. Given that chromosomal recombination only occurs in the male silkworm, to determine the chromosomal position of the locus controlling the response, the BF_1_ offspring from the male F_1_ and the female Sawa-J·*lem* were prepared. The silkworms were reared from hatching to the third instar on a commercial artificial diet containing mulberry leaf powder (Nosan Corporation, Yokohama, Japan) at 25°C, and the larvae were raised on fresh mulberry leaves from the fourth instar.

### 2. Electrophysiological Recordings

Fifth instar larvae, which had been feeding for one day, were used for the electrophysiological recordings. Taste responses were recorded from the lateral and medial styloconic sensillum on the maxillary galea using a classical tip-recording technique [8], [25]; however, we modified the method by recording from the intact living larva. The larva was immobilized inside a silicon tube (0.9 cm i.d.×5 cm) that had a longitudinal slit of approximately 1 cm in length from the edge. The head of the larva was taken out and pinched in the slit like a pillory on the neck, and the maxilla was kept protuberant by rolling with thin (3–5 mm wide) plastic paraffin film (Parafilm® M) on both the distal and proximal region alongside the maxilla. The sharpened tip of a stainless steel needle was inserted into the proximal region of the maxilla for use as an indifferent electrode. Glass capillaries with a tip diameter of 15–20 µm were used as recording/stimulating electrodes. A platinum wire dipped in the stimulus solution inside the capillary was connected to a TastePROBE amplifier (Syntech, Kirchzarten, Germany). The tip of the sensillum was capped with the capillary using a micromanipulator to record the response of taste neurons in the sensillum. Electrical signals were sampled and digitized with an IDAC-2 A/D converter (Syntech) and analyzed using AutoSpike software (Syntech). For each recording, a single sensillum was stimulated for 2 s, and the number of spikes generated 0.05–1.05 s after contact with the sensillum was counted. Successive recordings were conducted at least 2 min later to minimize the effect of adaptation to the neuronal response. Tissue paper was gently applied to the electrode tip just prior to each recording to minimize the effect of solvent evaporation at the recording/stimulating electrode tip. Most recordings were strong with regular firing and similar amplitude spikes, which meant that spikes were elicited from a single cell; however, some recordings possessed spikes with different small amplitudes. Since most small spikes are elicited from salt-sensitive cells [8], [9], [22], only the number of large spikes was counted, as these were the signals from the sucrose-best and inositol-specific cells and from the deterrent cell. Three to six different larvae were used for each concentration to elucidate dose–response relationships for the taste stimuli, and the average number of spikes and their standard errors were calculated.

### 3. Taste Stimuli

The sucrose-best and inositol-specific cells of the lateral styloconic sensilla in the two strains, Sawa-J·*lem* and Daiankyo, and their F_1_ progeny were tested for their responses to different concentrations (0.08, 0.4, 2, 10, 50, 250 mM) of sucrose and *myo*-inositol, which were dissolved in 4 mM NaCl as an electrolyte solution. The deterrent cell of the medial styloconic sensilla in Sawa-J·*lem*, Daiankyo, and the F_1_ were tested for their responses to both salicin (0.1, 1, 10, 25, 100 mM) and strychnine nitrate (0.0001, 0.001, 0.01 mM). We used 20 mM NaCl as an electrolyte solution to obtain a clear result on the responsiveness of the deterrent cell because a water taste neuron is associated with the same sensillum and its activity is depressed by mixing a higher concentration of salts [9]. A stimulant solution of 25 mM salicin was used to record from individual BF_1_ progeny larvae. All chemicals used as stimuli were purchased from Wako Pure Chemical Industries, Ltd. (Osaka, Japan).

## Results

**Figure 2:**
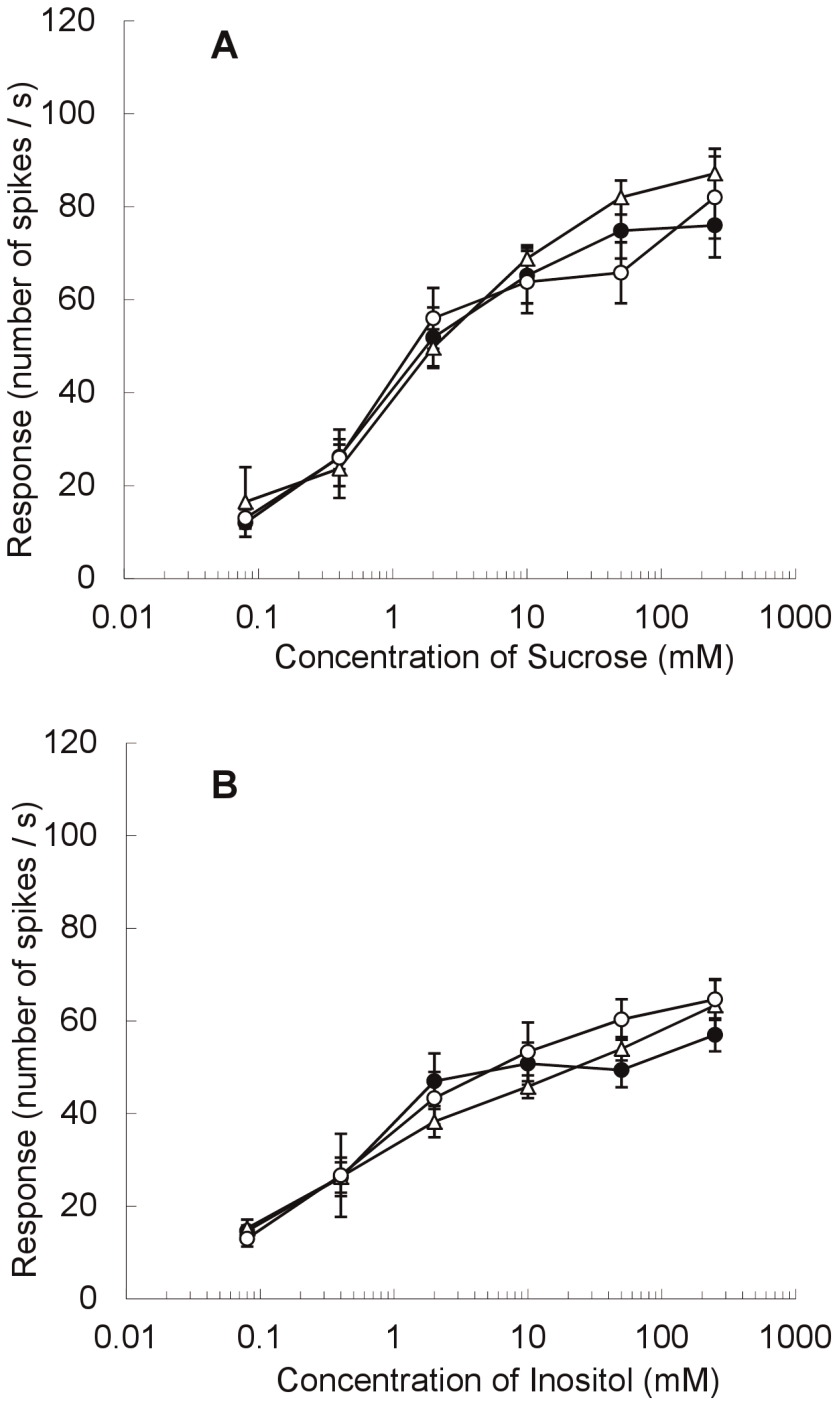
Dose–response to sucrose and inositol recorded from the lateral maxillary styloconic sensillum. Sensory response of the sucrose-best (A) and inositol-specific (B) taste cells in the lateral styloconic sensillum on the maxillary galea to different concentrations of sucrose and *myo*-inositol in the polyphagous silkworm Sawa-J·*lem* (•), normal silkworm Daiankyo (○), and their F_1_ progeny (△). The response was quantified by the number of spikes generated 0.05–1.05 s after the onset of stimulation. Values are shown as the average of three to six different larvae and the standard error. According to the result of Tukey’s HSD test (α = 0.05), no statistical significance was found in the values among the two parent strains and their F_1_ progeny.

**Figure 3:**
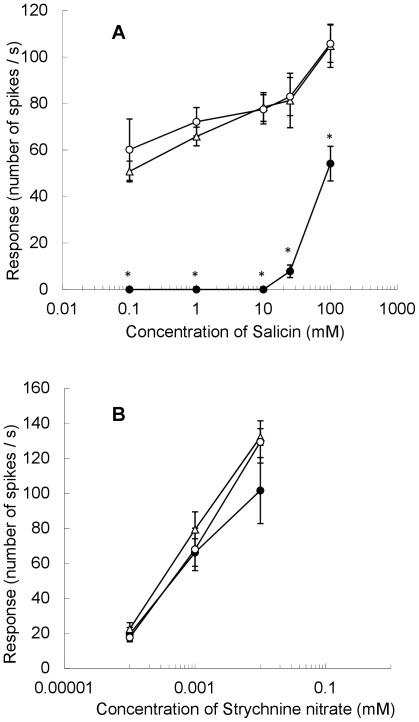
Dose–response to salicin and strychnine nitrate recorded from the medial maxillary styloconic sensillum. Sensory response of the deterrent cell in the medial styloconic sensillum on the maxillary galea to different concentrations of salicin (A) and strychnine nitrate (B) in the polyphagous silkworm Sawa-J·*lem* (•), normal silkworm Daiankyo (○), and their F_1_ progeny (△). The response was quantified by the number of spikes generated 0.05–1.05 s after the onset of stimulation. Values shown are the average of four to six different larvae and the standard error. The asterisks indicate the values of Sawa-J·*lem* that were significantly different from those of Daiankyo and the F_1_ progeny in Tukey’s HSD test (α = 0.05).

**Figure 4:**
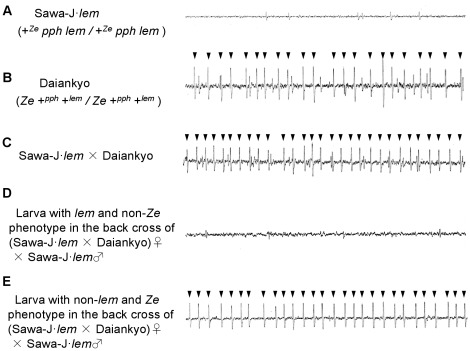
Representative responses of the deterrent cell to 25 mM salicin in larvae with different genotypes. Representative sensory responses of the deterrent cell in the medial styloconic sensillum on the maxillary galea to 25 mM salicin in the polyphagous silkworm Sawa-J·*lem* (A), normal silkworm Daiankyo (B), their F_1_ progeny (C), and *lem*- and non-*Ze*- phenotypes in the BF_1_ generation (D), and non-*lem* and *Ze* phenotypes in the BF_1_ generation (E). Because an F_1_ female was used in the backcross, crossing-over among the genes did not occur in the BF_1_ individuals. Therefore, larvae with *lem* and non-*Ze* phenotypes possessed the genotype of *lem pph* +*^Ze^/lem pph* +*^Ze^*, and larvae with non-*lem* and *Ze* phenotype were +*^lem^* +*^pph^ Ze/lem pph* +*^Ze^*. Recordings were performed using 10 larvae with the same genotype, and it was confirmed that they showed similar patterns. Each response trace shows a duration of 0.3 s beginning 0.5 s after the onset of stimulation. Arrowheads in B, C, and D indicate spikes from the deterrent cell.

To confirm that the Sawa-J·*lem* and Daiankyo strains used in the experiment possessed the same electrophysiological characteristics as those of the original Sawa-J and normal silkworm strains, the dose–responses of the sucrose-best and inositol-specific cells in the lateral styloconic sensilla were examined at different concentrations of sucrose and *myo*-inositol. As shown in Figure 2, Sawa-J·*lem*, Daiankyo, and their F_1_ progeny showed normal dose–response relationships for these taste stimuli, and no difference was observed among the responses in the two strains or their F_1_ larvae. We also recorded the dose–response of the deterrent cell against salicin and strychnine nitrate in the medial styloconic sensillum of Sawa-J·*lem,* Daiankyo, and their F_1_ progeny (Fig. 3). Although the number of spikes in response to 0.01 mM strychnine nitrate in Sawa-J·*lem* was slightly lower than that of Daiankyo and the F_1_, no marked difference was observed (Fig. 3, Fig. S1). In contrast, we found a significant difference in the responses to salicin among the two strains and the F_1_. The deterrent cell of Sawa-J·*lem* did not respond to salicin at concentrations of 0.1, 1.0, or 10 mM, and the response was very weak even at 25 mM, whereas Daiankyo and their F_1_ larvae clearly responded to 0.1 mM salicin, and the number of spikes increased with increasing concentration. Representative responses to 25 mM salicin in these silkworms are shown in Figure 4A–C, in which the deterrent cell responded with relatively larger spikes with regular intervals in Daiankyo and the F_1_ (arrowheads in Fig. 4B, C). Thus, the strains possessed the same electrophysiological characteristics of the original Sawa-J and normal strains for the taste responses reported previously [22]; i.e., maxillary taste neurons of the Sawa-J·*lem* and Daiankyo strains had normal dose–response relationships for sucrose, inositol, and strychnine nitrate, but the deterrent cell of Sawa-J·*lem* showed remarkably low sensitivity to salicin, in a manner similar to that of the original Sawa-J strain.

To investigate whether the low sensitivity to salicin in the two strains was caused by a single gene mutation in the *pph* locus, we first constructed two types of silkworms with the genotypes *lem pph* +*^Ze^/lem pph* +*^Ze^* and +*^lem^* +*^pph^ Ze/lem pph* +*^Ze^* in a cross of (Sawa-J·*lem* × Daiankyo)♀ × Sawa-J·*lem*♂ (Fig. 1B). Crossing-over does not occur in female chromosomes of silkworms. Therefore, a BF_1_ larva with the *lem* phenotype (yellow integument) is homozygous for *pph* as well as *lem*, and a larva with the *Ze* phenotype (zebra stripe integument) is heterozygous for *pph* as well as *Ze*. In the population of BF_1_ used for measuring the neural response to salicin, 441 eggs laid by one BF_1_ female were used. Among them, 423 larvae hatched, and the numbers of *lem* and *Ze*-phenotype larvae that survived to the fifth instar were 208 and 206, respectively. The values indicated that the genes related to the phenotypes and neural response to salicin did not affect viability during embryonic or larval development. We randomly selected 10 larvae with each of the two phenotypes and recorded their response to 25 mM salicin from the medial styloconic sensilla. All larvae with the *lem* phenotype showed almost no response and only small infrequent spikes (Fig. 4D), which was similar to the response pattern in the parent, Sawa-J·*lem* (Fig. 4A), whereas the *Ze-*phenotype larvae gave a clear response to 25 mM salicin with large spikes (arrowheads in Fig. 4E). These results indicated that the gene responsible for the different responses to 25 mM salicin is located on the same third chromosome as the *lem* and *Ze* genes. We then measured the frequency of crossing-over between the salicin response trait with *lem* and *Ze*. To obtain the crossover types among the loci, we crossed a Sawa-J·*lem* female and an F_1_ male between Sawa-J·*lem* and Daiankyo and counted the number of larvae with the *lem* and *Ze* characters in the BF_1_ progeny (Fig. 1B). The segregation ratio of the *lem* and *Ze* phenotypes is shown in Table 1. The frequency of crossover types between *lem* and *Ze* agreed with the theoretical ratio from the location of *lem* (3–0.0) and *Ze* (3–20.8) loci on the standard linkage map (http://www.shigen.nig.ac.jp/silkwormbase/ViewAllLinkageMap.do). Fifteen larvae were then randomly selected from the population with the normal integument phenotype and 15 larvae were selected with both the *lem* and *Ze* phenotypes as crossover types. We then tested these larvae for the electrophysiological response of the deterrent cell in the medial styloconic sensillum to salicin and counted the number of larvae with a normal reaction (positive response) and a weak reaction (negative response) to 25 mM salicin (Table 1). Nineteen larvae showed crossing-over between the *lem* locus and the gene controlling the response to salicin, and 11 larvae showed that crossing-over had taken place between the gene and *Ze*. Given a distance of 20.8 cM between *lem* and *Ze*, the locus of the gene controlling the response to salicin was calculated to be at 13.2 cM on the third chromosome. A chi-square analysis for the 30 crossing-over larvae showed no significant difference (*p* = 0.97) between the position of the locus of the gene controlling the electrophysiological response and the *pph* locus that was reported [21] at 12.9 cM on the third chromosome. According to the same test at the level of α = 0.05, the gene is located on the locus between 8.9 cM and 16.8 cM, and about 100 putative genes were found in the China gene model of the *B. mori* genome database (KAIKObase, http://sgp.dna.affrc.go.jp/KAIKObase/index.html).

**Table 1:** The number of larvae with positive and negative responses to 25 mM salicin in the BF_1_ progeny from a cross between Sawa-J·*lem* females and F_1_ males.

			Response to 25 mM salicin		
Phenotype		Number of larvae	Number of larvae tested	Positive	Negative
*lem*	+*^Ze^*	184	N.D.*	N.D.	N.D.
+*^lem^*	*Ze*	189			
+*^lem^*	+*^Ze^*	45	15	6	9
*lem*	*Ze*	46	15	10	5

The larvae with the *lem +^Ze^* and *+^lem^ Ze* phenotypes are non-crossover types.

The larvae with the *+^lem^ +^Ze^* and *lem Ze* phenotypes are crossover types.

* N.D., not determined.

## Discussion

The domesticated silkworm, *B. mori*, is a monophagous insect: it eats and grows on mulberry leaves and only a few other closely-related plant species. Genetic evidence indicates that its feeding preference behavior is controlled by many different genes. Several mutants, such as Nonpreference (Np), Nonpreference Shokei (Nps), Beet feeder (Bt), D^5X^, and Sek [18], [26], have a broad host range and consume different plants or a basic artificial diet without mulberry leaf powder; *Np*, *Nps*, *Bt*, and *Sek* are located on chromosomes 11, 3, 1, and 5, respectively. In contrast, the opposite type of feeding preference mutant was reported as ‘not feeding on an artificial diet’, *nfad* [27]. In addition to these genes, one locus controlling feeding preference behavior in the silkworm has been reported and designayed *pph* [12], [20]–[24]. The Sawa-J strain is a polyphagous strain bred from many geographical races of silkworms showing abnormal feeding behavior [19]. Sixty-six geographical races were screened for their feeding habit, and about 60 larvae were collected as a pool showing an abnormal feeding habit, such as eating cabbage or beet leaves. The Sawa-J strain was then created by repeated selection of the larvae showing abnormal feeding behavior. The breeding process of the Sawa-J strain suggests that the strain possesses many different genes allowing the ingestion of a wide variety of plants. Nevertheless, genetic analysis of the polyphagous character of Sawa-J showed that the feeding characteristic is controlled by a major gene(s) on the *pph* locus along with other, less potent genes [20].

Electrophysiological study of the *pph* mutant showed that the sensitivity of the taste neurons in the maxillary styloconic sensilla to inositol, sucrose, strychnine nitrate, and some other alkaloids, such as nicotine, brucine, and caffeine, was not much different from that of the normal silkworm, but the sensitivity to salicin as well as phloridzin, coumarin, and arbutin was much lower in Sawa-J compared with that of the normal silkworm [22]. This suggests that multiple signaling pathways are involved in the response to different bitter substances, which has also been proposed for *M. sexta* larvae on the basis of electrophysiological and behavioral analysis [15], [28]. In the closely related monophagous lepidopterous larvae, *Yponomeuta cagnagellus* and *Y. malinellus*, analysis of F_1_ progeny revealed that gustatory sensitivities to dulcitol, phroridzin, and prunasin are controlled by genetically dominant factors [29]. Few other genetic studies have examined taste sensitivity and the molecular mechanisms underlying taste transduction and recognition in lepidopteran larvae, which is in contrast to the wealth of physiological characterizations of the taste neurons and related studies on host-selection behavior [1], [2].

Our experiment was performed to determine whether the low sensitivity to salicin in the Sawa-J strain is controlled by the gene(s) on the *pph* locus. To perform the experiment, we marked the chromosome bearing the *pph* locus with a visible mutant gene, *lem*, and created the Sawa-J·*lem* strain. The new strain possessed the same electrophysiological taste response characteristics as the original Sawa-J strain. The F_1_ larvae of Sawa-J·*lem* and the normal strain Daiankyo recovered sensitivity of the deterrent cell to salicin. The results of a genetic analysis showed that this character was likely controlled by a single recessive gene because the ratio of larvae with normal and weak reactions to salicin in the BF_1_ of Sawa-J·*lem* and Daiankyo was about 1∶1 [see the results of (Sawa-J·*lem* × Daiankyo)♀ × Sawa-J·*lem*♂ and Table 1]. The locus of the gene controlling the sensitivity to salicin was determined from the number of crossover-type larvae (+*^lem^* +*^Ze^* and *lem Ze*) showing positive or negative electrophysiological reactions; it was located at a position nearly identical to *pph*, which was previously assessed by feeding behavior [21]. Thus, our genetic analysis suggested that *pph* and the locus of the gene controlling the sensitivity of the deterrent cell to salicin are at the corresponding position on the third chromosome, suggesting that *pph* may play a role in the salicin taste sensation and most likely for some other deterrent substances in the normal silkworm. Given the relatively small sample size (Table 1), it is possible that the *pph* locus corresponds to a set of tightly linked genes with similar or overlapping functions. Additionally, even as a single gene, *pph* may have as-yet unknown pleiotropic effects beyond the mechanism underlying the polyphagous behavior of the Sawa-J strain.

Molecular-level positional cloning and functional identification of *pph* are ongoing in our laboratory. Monogenic controls of taste sensitivity and feeding behavior were reported exclusively for seven-transmembrane gustatory receptor proteins (Grs) in *Drosophila melanogaster* [30]–[34]. Among these, 33 Grs appear to be expressed in labellar bitter neurons [35]. Two odorant binding proteins (OBPs) that express in the tarsal taste sensilla of *Drosophila* species and presumably interact with two plant fatty acids are responsible for host-plant preference behavior [36], [37]. However, so far, neither Grs nor OBPs were identified in the chromosome regions for the *pph* locus delimited by SNP mapping (T. Iizuka, personal communication) using the *B. mori* genome database (KAIKObase). On the other hand, a TRP channel with a downstream of phospholipase C (PLC)-dependent signaling cascade is involved in taste sensation to aristolochic acid but not to other bitter substances in *D. melanogaster* [38]. Signaling cascades of the intracellular second messengers, 3′,5′ cyclic guanosine monophosphate (cGMP) and inositol 1,4,5-triphosphate (IP_3_), appear to be involved in the transduction or modulation of bitter taste sensation in flies [39], [40]. The genes regulating these pathways or other unknown genes are candidates for *pph.*

## Supporting Information

Figure S1 **Representative responses of the deterrent cell to 0.01 mM strychnine nitrate in larvae with different genotypes.** Representative sensory responses of the deterrent cell in the medial styloconic sensillum on the maxillary galea to 0.01 mM strychnine nitrate in the polyphagous silkworm Sawa-J·*lem* (A), normal silkworm Daiankyo (B), and their F_1_ progeny (C). The experiment to determine the representative response was performed using 5–10 larvae with the same genotypes and it confirmed that they showed the same pattern. Each response trace shows a duration of 0.3 s beginning 0.5 s after the onset of stimulation.
(TIF)
