# Supplementary figures and images for: Genetic Analysis of the Electrophysiological Response to Salicin, a Bitter Substance, in a Polyphagous Strain of the Silkworm Bombyx mori
Source: PLoS One. 2012 May 23;7(5):e37549. doi: 10.1371/journal.pone.0037549 (PMC3359296; doi:10.1371/journal.pone.0037549)

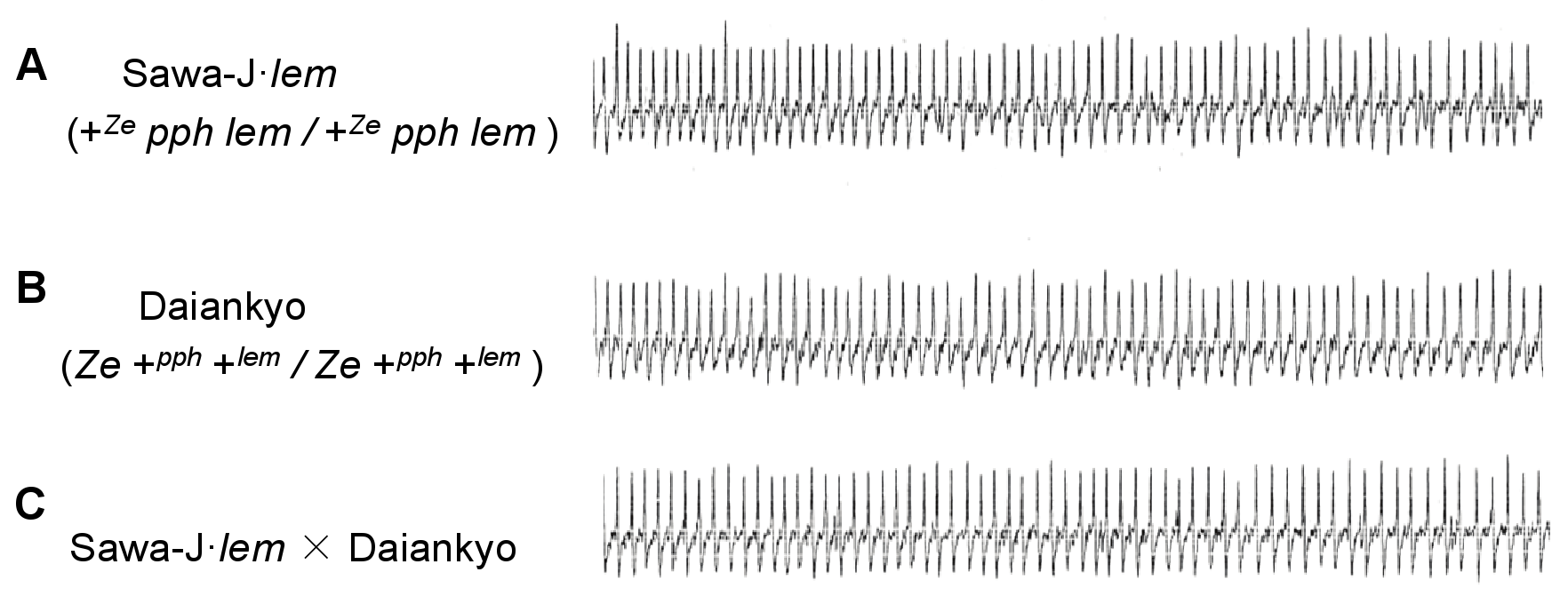

Supplement: Figure S1 — Representative responses of the deterrent cell to 0.01 mM strychnine nitrate in larvae with different genotypes. Representative sensory responses of the deterrent cell in the medial styloconic sensillum on the maxillary galea to 0.01 mM strychnine nitrate in the polyphagous silkworm Sawa-J·lem (A), normal silkworm Daiankyo (B), and their F1 progeny (C). The experiment to determine the representative response was performed using 5–10 larvae with the same genotypes and it confirmed that they showed the same pattern. Each response trace shows a duration of 0.3 s beginning 0.5 s after the onset of stimulation. (TIF) [file pone.0037549.s001.tif]
